# Prognostic Relevance of the Peritoneal Surface Disease Severity Score Compared to the Peritoneal Cancer Index for Colorectal Peritoneal Carcinomatosis

**DOI:** 10.1155/2016/2495131

**Published:** 2016-02-23

**Authors:** Jia Lin Ng, Whee Sze Ong, Claramae Shulyn Chia, Grace Hwei Ching Tan, Khee-Chee Soo, Melissa Ching Ching Teo

**Affiliations:** ^1^Department of Surgical Oncology, National Cancer Centre Singapore, 11 Hospital Drive, Singapore 169610; ^2^Division of Clinical Trials & Epidemiological Sciences, National Cancer Centre Singapore, 11 Hospital Drive, Singapore 169610

## Abstract

*Background*. Peritoneal Carcinomatosis Index (PCI) is a widely established scoring system that describes disease burden in isolated colorectal peritoneal carcinomatosis (CPC). Its significance may be diminished with complete cytoreduction. We explore the utility of the recently described Peritoneal Surface Disease Severity Score (PSDSS) and compare its prognostic value against PCI.* Methods.* The endpoints were overall survival (OS), progression-free survival (PFS), and survival less than 18 months (18 MS).* Results.* Fifty patients underwent cytoreductive surgery and hyperthermic intraperitoneal chemotherapy (CRS/HIPEC) for CPC from 2003 to 2014, with 98% achieving complete cytoreduction. Median OS was 28.8 months (95% CI, 18.0–39.1); median PFS was 9.4 months (95% CI, 7.7–13.9). Univariate analysis showed that higher PCI was significantly associated with poorer OS (HR 1.11; 95% CI, 1.03–1.20) and PFS (HR 1.09; 95% CI, 1.03–1.14). Conversely, PSDSS was not associated with either endpoint. Multivariate analysis showed that PCI, but not PSDSS, was predictive of OS and PFS. PCI was also able to discriminate survival outcomes better than PSDSS for both OS and PFS. There was no association between 18 MS and either score.* Conclusion.* PCI is superior to PSDSS in predicting OS and PFS and remains the prognostic score of choice in CPC patients undergoing CRS/HIPEC.

## 1. Introduction

Cytoreductive surgery and hyperthermic intraperitoneal chemotherapy (CRS/HIPEC) have resulted in improved survival outcomes for patients with isolated colorectal peritoneal carcinomatosis (CPC) [1–4]. Improved operative morbidity and mortality, that is, on a par with liver resections for isolated colorectal liver metastases, have also contributed to the increasing acceptance of this treatment modality [5]. The Peritoneal Cancer Index (PCI) and Completeness of Cytoreduction (CC) scores, as described by Jacquet and Sugarbaker [6], aid in predicting postoperative survival outcomes. However, in the presence of optimal cytoreduction regardless of PCI score, one wonders if the prognostic significance of PCI may be rendered irrelevant.

First described by Sugarbaker in 1998 [7], the Peritoneal Surface Disease Severity Score (PSDSS) incorporates clinical symptoms and primary tumour histology with the PCI. There is a growing interest in PSDSS after studies showed its utility in prognostication. However, there is a paucity of data directly comparing it to the more established PCI used in many institutions, including ours. Therefore, the aim of this study was to compare the predictive value of PCI versus PSDSS in our study population where complete cytoreduction was almost always achieved.

## 2. Materials and Methods

### 2.1. Patient Selection

In this Singapore Health Services Ethics Board approved study, a retrospective review of a prospectively maintained database was performed for patients who had undergone CRS/HIPEC for peritoneal carcinomatosis from colorectal cancer. As the only tertiary centre offering CRS/HIPEC in South East Asia, these surgeries performed between February 2003 and April 2014 were carried out by two surgeons with special interest in advanced surgical oncology and CRS/HIPEC, with the second surgeon beginning midway into the period of analysis. CRS/HIPEC performed for appendiceal malignancies and other noncolorectal malignancies were excluded. Cases in which complete cytoreduction was deemed not feasible during exploratory laparotomy, and in which CRS/HIPEC was eventually not performed, were also excluded from analysis. Patients were routinely followed up closely with clinical examination and tumour markers every 3 months, and radiological imaging at least every 6 months, at the discretion of the treating surgeon.

### 2.2. Prognostic Scores

Peritoneal Cancer Index (PCI) and the Peritoneal Surface Disease Severity Score (PSDSS) were calculated and compared. PCI was calculated according to lesion size and its distribution in 9 abdominopelvic regions and 4 small bowel segments noted intraoperatively [6]. During exploratory laparotomy, patients in whom optimal cytoreduction was not deemed possible had their planned CRS/HIPEC procedure abandoned. Optimal cytoreduction in invasive cancers like CRC is defined as achieving a CC score of 0 to 1, with CC-0 indicating no macroscopic residual disease and CC-1 indicating no residual nodules greater than 2.5 mm. The prognosis for suboptimal or incomplete cytoreduction is universally dismal, with the risks of undertaking further morbid surgery far greater than any potential therapeutic gains [7]. PSDSS consists of 3 prognostic categories: clinical symptoms, primary tumour pathology, and PCI score [8], each of which is subcategorized according to severity. The endpoints used were overall survival (OS), progression-free survival (PFS), and survival less than 18 months (18 MS), as most patients survived beyond that. Demographic data and surgical outcomes were also obtained.

### 2.3. Statistical Analysis

A total of 61 laparotomy procedures were performed, but 7 did not complete CRS/HIPEC as the volume of disease determined intraoperatively was not found to be amenable for optimal CRS. In total, 54 CRS/HIPEC operations were completed on 51 patients. Three patients had redone CRS/HIPEC, and only their first operative records were selected for analysis. In addition, one patient with a PCI score of zero was excluded from the study. A total of 50 patients were analysed for OS and PFS. For the 18 MS analysis, 24 patients who were still alive or who were lost to follow-up before 18 months were further excluded.

PCI was analysed both as a continuous variable and as a categorical variable with 3 levels (<10, 10–20, and >10). PSDSS was also analysed as a continuous variable and as a categorical variable with 4 levels (<3, 4–7, 8–10, and >10). Categorical cut-offs for analysis were based on evidence from similar recently published prognostication studies [9–12].

OS was calculated from the date of surgery to the date of demise from any cause. PFS was calculated from the date of surgery to the date of disease relapse or demise, whichever occurred first. All survival distributions were estimated using Kaplan-Meier curves, and the log-rank test was used to test differences between curves. Cox proportional hazard regression models were fitted to estimate hazard ratios. For multivariate analysis, only PCI and PSDSS were fitted in the Cox model after considering the relatively small number of events for OS and PFS and that the primary objective of the study was to compare the prognostic value of PCI against PSDSS. Proportional hazards assumption was verified for each fitted model using Schoenfeld residuals. The discriminative ability of each prognostic score for OS/PFS was evaluated based on Harrell's concordance index for censored data (*c*-index) [13] and the *D*-statistics (*D*-stats) of Royston and Sauerbrei [14]. The *c*-index represents the probability of concordance between predicted and observed survival, taking value from 0.5 (random prediction) to 1 (perfect ability to discriminate). The *D*-stats measure a prognostic score's ability to separate the risk of death and/or relapse. The larger the *D*-stats, the greater the degree of separation for a prognostic score.

Logistic regression models were fitted to estimate the odds ratios to assess the association of various variables with 18 MS. The ability of the prognostic score to correctly classify patients who were dead or not within 18 months after CRS/HIPEC was evaluated based on the area under the receiver operating characteristics curve (AUC). The AUC takes value from 0.5 (random prediction) to 1 (perfect discrimination ability).

Statistical significance was set at *p* < 0.05. Analyses were performed using SAS version 9.4 (SAS Institute Inc., Cary, NC) with the *D*-stats generated using STATA 12.0 (Stata Corp, College Station, TX).

## 3. Results

Demographic data and surgical outcomes of the 50 patients were summarised in Table 1. The median age was 50 years (range, 14–71), and Eastern Cooperative Oncology Group performance status was 0 (82.0%) or 1 (18.0%). CC-0 score was achieved in 98% of cases, with only one case of moderately differentiated adenocarcinoma achieving CC-1 score. The median operative time was 457.5 minutes (range, 120–960). All patients underwent HIPEC (intraperitoneal mitomycin C or oxaliplatin, leucovorin and 5-fluorouracil combination, at a temperature of 39 to 43 degrees Celsius for 60 to 90 minutes). Twenty-four patients underwent early postoperative intraperitoneal chemotherapy, which was within the treatment protocol before October 2012. The median hospitalisation duration was 13.5 days (range, 9–43), and 22% of patients received adjuvant therapy after surgery at the discretion of the treating physician.

The median PCI was 10 (range, 1–27), and the median PSDSS was 6 (range, 2–22). In terms of cancer grade, 54.0% of patients had moderately differentiated carcinoma, 26.0% had poorly differentiated or mucinous carcinoma, and 2.0% had signet ring cell carcinoma.

The median follow-up duration was 13.3 months (range, 0.8–87.1 months) (Table 2). The median OS was 28.8 months (range, 18.3–39.1) with 87.6% surviving at 1 year. The median PFS was 9.4 months (range, 7.7–13.9), and 1-year PFS was 37.6%.

The results of univariate Cox-regression analysis, *D*-stats, and *c*-index for both PCI and PSDSS as continuous variables are summarised in Table 3. Higher PCI values were significantly associated with poorer OS (hazard ratio (HR) = 1.11; 95% CI, 1.03–1.20; and *p* = 0.005). PSDSS was not associated with OS (HR = 1.06; 95% CI, 0.97–1.14; and *p* = 0.191). Based on *D*-stats and *c*-index, PCI had higher discrimination ability than PSDSS for OS. On multivariate Cox analysis by PCI and PSDSS, only PCI was shown to be an independent significant predictor for OS (HR = 1.16; 95% CI 1.04–1.30; and *p* = 0.008) (Table 4).

As for PFS, higher PCI values were significantly associated with poorer PFS (HR = 1.09; 95% CI, 1.03–1.14; and *p* = 0.001). There was no significant association between PFS and PSDSS (HR = 1.05; 95% CI 0.99–1.11; and *p* = 0.096). PCI had a higher discrimination ability than PSDSS for PFS, with higher *D*-stats and *c*-index scores. On multivariate analysis, PCI was again shown to be an independent significant predictor for PFS (HR = 1.11; 95% CI 1.03–1.20; and *p* = 0.005).

Neither PCI nor PSDSS had any significant association with 18 MS (PCI: odds ratio (OR) = 1.14; 95% CI, 0.99–1.31 versus PSDSS: OR = 1.03; 95% CI, 0.89–1.19). While PCI had higher AUC than PSDSS (0.742 versus 0.585), there was no significant difference between both AUCs (difference = 0.157; 95% CI, −0.03–0.34; and *p* = 0.091).

## 4. Discussion

Prognostication scores have gained interest in the field of colorectal cancer with isolated peritoneal carcinomatosis in recent years [8–12]. The ideal score is easy to apply in clinical practice and backed by robust evidence in predicting overall survival and disease progression. Some studies have begun formulating and evaluating scores to predict resectability prior to surgery [9–12].

One such score is the PSDSS. This study explored the utility of using PSDSS in lieu of intraoperative PCI as a prognostic score. Nonetheless, we have found the PCI to be superior to PSDSS in prognosticating overall survival and disease progression. The inclusion of tumour biology and patient symptomology did not aid in discriminating outcome among our patients. This may be due to all but 1 patient achieving macroscopic complete cytoreduction (CC-0), regardless of disease burden. Our commitment to eradicate all disease in the peritoneal cavity, no matter how minute, may have rendered this additional information in the scoring system superfluous.

Furthermore, histopathology of the primary tumour plays a less definitive role in CPC. A French multicentre study [15] showed that positive independent prognostic factors include PCI, completeness of surgery, lymph node status, experience of the centre, and the use of adjuvant chemotherapy. Tumour grade did not appear to be significantly related to overall survival or progress-free survival.

Most of the patients developed progressive disease with interval development of CPC after the initial colorectal surgery. With many on regular postoperative surveillance and early referrals from other practitioners, not many develop symptoms before their early diagnosis of peritoneal carcinomatosis. With 32 of 50 patients asymptomatic on presentation, the inclusion of patient symptomology in PSDSS may not have helped in discriminating for outcome.

Neither PCI nor PSDSS was able to predict 18-month survival. The results may be limited by the relatively small number of patients available for analysis and those who survived less than 18 months, resulting in few measured events.

Surgical treatment of peritoneal metastases has gained momentum from oncologic communities around the world in the past decade. Initially viewed as an advanced disease doomed for palliative chemotherapy, referrals to the surgeons were scanty in the early 2000s and only began to pick up from 2010 onwards. The indications for CRS/HIPEC for colorectal peritoneal carcinomatosis have also broadened in recent years, compared to the initial years when the program initially started. We are in the midst of performing a learning curve analysis for all cases, but it is not inconceivable that our results are typical of any institution with the introduction of a complex procedure.

A potential drawback to the current study is the small sample size, contributed by the factors mentioned above, with a heterogeneous population, as typical of a retrospective analysis of our experience. The cut-offs for categorical analysis of PCI and PSDSS were based on evidence from recently published prognostication studies. While the precision of these analyses was affected by the small sample in some of the categories, it is essential to allow for meaningful comparisons across other similar publications comparing the two prognostic scores [9–12]. We performed sensitivity analyses to evaluate the effect of small PCI and PSDSS categories. The conclusion remained similar when the small categories with less than 10 patients were combined with larger categories (results not shown).

Moving forward, we will continue to use intraoperative PCI as a tool for prognostication for overall survival and progression-free survival. With an increasing acceptance of CRS/HIPEC in treating colorectal peritoneal carcinomatosis, improving chemotherapy options and enhanced learning curves, one can look forward to a larger sample size with enhanced outcomes.

With that, we will continue to compare the utility of prognostic scores like PSDSS, and even the COREP score [9], which includes tumour markers to attempt to prognosticate outcomes and predict feasibility of achieving CC-0 resections prior to surgery.

We have also embarked on comparing preoperative PCI and PSDSS based on interpretation of radiological findings and are exploring the utility of radiologically calculated PCI and PSDSS for all patients considered for CRS and HIPEC. CT-PCI has been shown to have a sensitivity of 0.55 and a specificity of 0.86, compared to MRI-PCI with a sensitivity of 0.95 and specificity of 0.70 in a recently published paper comparing these scores amongst patients with appendiceal and ovarian cancer [16]. These adjunct imaging modalities may allow discrimination between those who would go on to have successful CRS and HIPEC and those in whom complete cytoreduction is not possible.

## 5. Conclusion

PCI remains the prognostic score of choice for patients with CPC undergoing CRS/HIPEC, as it is superior to PSDSS in prognosticating OS and PFS.

## Figures and Tables

**Table 1:** Baseline demographics and operative findings.

	Number	%

Total	50	100.0
Age at CRS/HIPEC, years		
Median (range)	50 (14–71)	
Gender		
Female	32	64.0
Male	18	36.0
ECOG performance status		
0	41	82.0
1	9	18.0
Presence of comorbidities		
No	19	38.0
Yes	31	62.0
Primary tumour site		
Colon	49	98.0
Rectum	1	2.0
Histology		
Well differentiated	6	12.0
Moderately differentiated	27	54.0
Poorly differentiated or mucinous	13	26
Signet ring cell	1	2.0
Missing data	3	6.0
Preoperation CEA, μg/L		
Median (range)	5.1 (0.2–501.0)	
Type of CRS procedure		
Subdiaphragmatic stripping	30	60.0
Gastrectomy	4	8.0
Colectomy	24	48.0
Small bowel resection	21	42.0
Splenectomy	6	12.0
THBSO	15	30.0
Cholecystectomy	8	16.0
Bladder resection	5	10.0
Others	17	34.0
Number of CRS procedures performed		
0	1^a^	2.0
1	14	28.0
2	18	36.0
3 and over	17	34.0
Median (range)	2 (0–5)	
Duration of operation, mins		
Median (range)	457.5 (120–960)	
Completion of Cytoreduction score		
0	49	98.0
1	1	2.0
≥2	0	0
Hospitalization duration, days		
Median (range)	13.5 (9–43)	
Post-CRS/HIPEC adjuvant therapy		
No	26	52.0
Yes	11	22.0
Missing data	13	26.0

CRS, cytoreductive surgery; HIPEC, hyperthermic intraperitoneal chemotherapy; ECOG, Eastern Cooperative Oncology Group; CEA, carcinoembryonic antigen; THBSO, Total Abdominal Hysterectomy Bilateral Salpingo Oophorectomy.

^a^The patient had omentectomy.

**Table 2:** Survival outcomes and relapses.

	Number	%
Follow-up duration, months		
Median (range)	13.3 (0.8–87.1)	
Overall survival (OS)		
Events/patients	18/50	
Median OS, months (95% CI)	28.8 (18.3–39.1)	
1-year OS, % (95% CI)	87.6 (72.7–94.7)	
Progression-free survival (PFS)		
Events/patients	33/50	
Median PFS, months (95% CI)	9.4 (7.7–13.9)	
1-year PFS, % (95% CI)	37.6 (22.7–52.5)	
*Among relapsed patients*	*32*	*100.0*
Site of relapse		
Peritoneum	28	87.5
Lymph nodes	11	34.4
Lung	15	46.9
Liver	8	25.0
Bones	5	15.6
Others	8	25.0

CI, confidence interval.

**Table 3:** Univariate Cox-regression analysis, *D*-stats, and *c*-index of PCI and PSDSS for OS and PFS.

	Overall survival (OS)						Progression-free survival (PFS)					
	E/N	Median OS, month	HR (95% CI)	*p* ^∧^	*D*-stats	*c*-index	E/N	Median PFS, month	HR (95% CI)	*p* ^∧^	*D*-stats	*c*-index
*PCI*												
Per unit increase	18/50	28.8	1.11 (1.03–1.20)	**0.005** ^#^	—	0.737	33/50	9.4	1.09 (1.03–1.14)	**0.001** ^#^	—	0.680
0 ⩽ PCI ⩽ 9	4/25	NR	1	0.059	1.033	0.693	10/25	17.6	1	**0.004**	0.870	0.668
10 ⩽ PCI ⩽ 20	11/20	27.1	3.23 (1.03–10.19)				19/20	7.2	3.45 (1.59–7.49)			
PCI > 20	3/5	19.9	4.55 (0.99–20.98)				4/5	7.8	2.85 (0.88–9.27)			
*PSDSS*												
Per unit increase	18/50	28.8	1.06 (0.97–1.14)	0.191^#^	—	0.613	33/50	9.4	1.05 (0.99–1.11)	0.096^#^	—	0.599
2 ⩽ PSDSS ⩽ 3	0/5	NR	1	0.139	0.686	0.603	0/5	NR	1	0.077	0.652	0.561
4 ⩽ PSDSS ⩽ 7	10/26	28.8	a				18/26	9.4	a			
8 ⩽ PSDSS ⩽ 10	2/5	18.5	a				3/5	8.4	a			
PSDSS > 10	6/14	34.5	a				12/14	9.4	a			

E, events; N, patients; HR, hazard ratio; CI, confidence interval; NR, not reached; PCI, Peritoneal Cancer Index; PSDSS, Peritoneal Surface Disease Severity Score.

^a^Not estimable as there were no events in the reference group.

^∧^Based on log-rank test, unless otherwise specified.

^#^Based on Wald's test.

**Table 4:** Multivariate Cox-regression analysis of OS and PFS by PCI and PSDSS.

	Overall survival (OS)		Progression-free survival (PFS)	
	HR (95% CI)	*p* ^#^	HR (95% CI)	*p* ^#^
PCI	1.16 (1.04–1.30)	**0.008**	1.11 (1.03–1.20)	**0.005**
PSDSS	0.93 (0.82–1.06)	0.271	0.97 (0.89–1.05)	0.403

HR, hazard ratio; CI, confidence interval; PCI, Peritoneal Cancer Index; PSDSS, Peritoneal Surface Disease Severity Score.

^#^Based on Wald's test.
